# Prenatal CFTR modulator therapy for fetal cystic fibrosis: Emerging evidence, clinical considerations, and future directions

**DOI:** 10.1002/pmf2.70317

**Published:** 2026-07-16

**Authors:** Hiba J. Mustafa, Yair J. Blumenfeld, Ramen H. Chmait, Jennifer L. Taylor‐Cousar, Jordana E. Hoppe, Stacey L. Martiniano, Angela Metcalf, Scott D. Sagel, Edith T. Zemanick, Alireza A. Shamshirsaz, Sylvia E. Szentpetery, Aaron T. Trimble, Elizabeth B. Burgener, Carmen Luna‐Paredes, Isabelle Sermet‐Gaudelus, Michael V. Zaretsky

**Affiliations:** ^1^ Division of Maternal‐Fetal Medicine The Fetal Center at Indiana University and Riley Children's Health Indianapolis Indiana USA; ^2^ Department of Obstetrics & Gynecology Division of Maternal‐Fetal Medicine and Obstetrics School of Medicine Stanford University Stanford California USA; ^3^ Department of Obstetrics and Gynecology Division of Maternal‐Fetal Medicine Keck School of Medicine University of Southern California Los Angeles California USA; ^4^ Departments of Medicine and Pediatrics National Jewish Health University of Colorado Anschutz Medical Campus Denver Colorado USA; ^5^ Department of Pediatrics Children's Hospital Colorado University of Colorado Anschutz Medical Campus Aurora Colorado USA; ^6^ Comprehensive Fetal Care Center at Dell Children's Dell Children's Medical Center Austin Texas USA; ^7^ Department of Pediatrics Medical University of South Carolina Charleston South Carolina USA; ^8^ Division of Pulmonary Allergy, and Critical Care Medicine Oregon Health & Science University Portland Oregon USA; ^9^ Department of Pediatrics Division of Pediatric Pulmonology & Sleep Medicine Children's Hospital Los Angeles Keck School of Medicine at University of Southern California Los Angeles California USA; ^10^ Department of Pediatrics Unidad Multidisciplinar Fibrosis Quística Hospital Universitario 12 de Octubre Madrid Spain; ^11^ Hospital Necker‐Enfants Malades, CF Center, Inserm U1151 Universite Paris Cite Paris France; ^12^ Department of Obstetrics and Gynecology Colorado Fetal Care Center University of Colorado School of Medicine Denver Colorado USA

**Keywords:** CFTR, cystic fibrosis, ETI, fetal therapy, meconium ileus, modulator therapy, prenatal therapy, Trikafta

## Abstract

**Background:**

The consequences of cystic fibrosis (CF) transmembrane conductance regulator (CFTR) protein dysfunction or absence begin during fetal development, with pancreatic, intestinal, hepatobiliary, and reproductive manifestations evident at birth. The advent of CFTR modulator therapies (CFTR modulator)—most notably elexacaftor/tezacaftor/ivacaftor (ETI), approved for children and adults with CF who carry responsive variants, and the recently approved vanzacaftor/tezacaftor/deutivacaftor (VTD), currently indicated for older pediatric and adult populations—has transformed long‐term health outcomes and substantially increased the number of pregnancies among individuals with CF. As maternal use of CFTR modulator during pregnancy has become more common, early observational studies and case series suggest potential fetal benefit for fetuses affected by CF, particularly with respect to inspissated meconium obstructing the terminal ileum, potentially reducing the need for postnatal surgical intervention.

**Methods:**

To synthesize current evidence regarding maternal, fetal, and neonatal outcomes following prenatal CFTR modulator exposure; describe the rationale and early experience with intentional prenatal therapy for affected fetuses; and outline practical counseling and monitoring strategies. Evidence was drawn from clinical pregnancy cohorts, pharmacokinetic studies, case series treating fetal meconium ileus, mechanistic fetal and animal models, and expert consensus documents, including the PRenatal MOdulator Treatment to PrEvent CF ComplicaTions (PROTECT) Workshop summary. Both maternal‐ and fetal‐indication uses of CFTR modulator were evaluated.

**Results:**

Continuation of ETI during pregnancy has been associated with stabilization of maternal pulmonary function and nutritional status and has not been linked to an increased risk of congenital anomalies, preterm delivery, or miscarriage in available observational cohorts. Both ETI and VTD cross the placenta and achieve fetal drug exposure at concentrations expected to be pharmacologically active. Case series in fetuses with CF describe improvement or resolution of prenatal bowel abnormalities indicative of evolving meconium ileus, with suggestive evidence of preservation of exocrine pancreatic function and the male reproductive tract in limited cases. Cataracts have rarely been reported, are typically small and not visually significant, and have not been consistently observed across cohorts. Prenatal exposure may result in falsely negative newborn screening results. However, the optimal timing and duration of prenatal modulator initiation for fetal benefit, fetal pharmacokinetics and pharmacodynamics, and long‐term neurodevelopmental outcomes remain uncertain.

**Conclusion:**

Prenatal CFTR modulator therapy shows promise as the first fetal therapy for CF. While continuation for maternal CF is supported by available evidence (although not label‐approved), fetal‐indication use remains investigational. Robust counseling, interdisciplinary coordination, and structured monitoring are essential, alongside ongoing research to define therapeutic windows, safety, and long‐term outcomes.

## 1 INTRODUCTION

Cystic fibrosis (CF) is a progressive, multisystem genetic disorder historically associated with high morbidity and limited lifespan. With the advent of CF transmembrane conductance regulator (CFTR) modulator therapy (CFTR modulator) for those who are genetically eligible, tolerate treatment, and have access to these medications, survival and quality of life have improved substantially [1]. Elexacaftor/tezacaftor/ivacaftor (ETI) and vanzacaftor/tezacaftor/deutivacaftor (VTD) further expand therapeutic coverage for individuals with responsive variants [2, 3]. Improved lung function, nutritional status, and reproductive health in females have contributed to an increase in pregnancies among individuals with CF [4].

Because most individuals with CF are receiving CFTR modulator and the benefit to maternal health is so substantial, most individuals with CF choose to continue modulators during pregnancy, making fetal exposure increasingly common. Retrospective pregnancy studies show stabilization or improvement in maternal pulmonary status and no increased risk of congenital anomalies. [5, 6] The Maternal and Fetal Outcomes in the Era of Modulators (MAYFLOWERS) study (NCT04828382) is an ongoing prospective multicenter observational study in pregnancies with CF evaluating maternal, fetal, and infant outcomes among pregnancies exposed to CFTR modulator therapy in pregnancies with CF as well as those who are carriers for a CFTR variant, including pharmacokinetics during pregnancy and lactation. The study aims to provide systematic safety data regarding modulator continuation during pregnancy and early infancy, the MODUL‐CF study (NCT04301856) is an ongoing prospective multicenter cohort evaluating the use of CFTR modulator during pregnancy, specifically for fetal indications, particularly treatment of fetuses with CF and ultrasound findings suggestive of meconium ileus. The study aims to characterize fetal imaging response, neonatal outcomes, and potential modification of early CF disease manifestations following prenatal therapy, and the PROTECT CF, which is a workshop initiative exploring CFTR modulator therapy during pregnancy to prevent CF complications in fetuses with CF, concluding that a prospective trial is warranted based on promising animal and human case data [7].

Historical evidence demonstrates that CF pathology begins in utero—affecting the pancreas, intestine, airways, and reproductive tract—providing a biologically compelling rationale for prenatal molecular therapy.

Emerging case reports and case series suggest that intentional prenatal use of ETI may improve CF fetal outcomes, particularly treatment and resolution of meconium ileus [8, 9, 10]. This use has led to increasing referrals to maternal‐fetal medicine (MFM) and CF specialists for counseling on prenatal diagnosis, therapeutic options, and fetal surveillance.

## 2 PATHOPHYSIOLOGY OF CF IN PRENATAL LIFE

### 2.1 Early fetal onset of disease

Fetal pathology studies demonstrate that CF organ manifestations begin well before birth. Pancreatic acinar destruction and ductal plugging are evident by the second trimester. Thickened meconium, reduced intestinal fluid secretion, and impaired peristalsis contribute to meconium ileus and bowel dilation. Airway surface liquid depletion, mucus accumulation, and ion transport defects appear in fetal airway models. Early male reproductive tract abnormalities, including vas deferens degeneration, also develop prenatally [11].

## 3 RATIONALE FOR PRENATAL CFTR MODULATOR THERAPY

### 3.1 Overview of CFTR modulators

CFTR modulator includes correctors, small molecules that improve folding and trafficking of the CFTR protein to the cell surface (tezacaftor, elexacaftor, and vanzacaftor) and potentiators, small molecules that improve ion transport through the CFTR protein present at the cell surface (ivacaftor, deutivacaftor) [3]. To date, the greatest benefit has been demonstrated when two correctors and one potentiator are used in combination. The combination therapies with the highest use (where available) are elexacaftor and tezacaftor in combination with ivacaftor (ETI) and vanzacaftor and tezacaftor in combination with deutivacaftor (VTD). ETI and VTD restore CFTR function to 40%–60% of wild‐type (normal) CFTR activity in vitro for approximately 90%–95% of people with CF in countries with predominantly northern European populations.

ETI and VTD are effective for individuals with at least one Phe508del (F508del) allele or other ETI/VTD‐responsive variants, provided no additional variant within a complex allele is present that inhibits ETI activity; VTD further expands the pool of modulator‐responsive variants, representing the majority of individuals affected with CF [3].

### 3.2 Genotype‐phenotype correlations and treatment implications

CF genotype strongly correlates with pancreatic status, with approximately 85% of individuals with CF having pancreatic insufficiency, typically those with “severe” or minimal function class I, II, or III CFTR variants [12]. Individuals with one “milder” or residual function class IV or V CFTR variant, even when paired with a minimal function variant, tend to be pancreatic sufficient [13]. Individuals with residual function variants associated with PS are generally at less risk for meconium ileus, compared with individuals with two minimal function variants [13].

Some genetic variants have varying clinical consequences that complicate treatment decisions. While certain variants may technically qualify for treatment with ETI based on in vitro responsiveness, the variant may not always cause disease. Thus, the clinical phenotype may be mild or even show no disease manifestations, and there is the risk of treating a fetus who will not have CF. These individuals with milder variants typically do not have pancreatic insufficiency or meconium ileus issues [14].

The U.S. Food and Drug Administration (FDA) expanded the ETI label to include 177 additional rare CFTR variants based on in vitro data, though clinical responses can vary significantly [15].

Having a team member who understands CF genetics is crucial for guiding treatment decisions. Genetic counselors can accurately identify and interpret CFTR variants, inform patients about individualized treatment options, and guide enrollment in clinical trials for gene‐based therapies. This expertise is particularly important given the rise of newborn screening with CFTR gene sequencing, which has identified more individuals with rare or complex variants requiring nuanced interpretation [16].

#### 3.2.1 Maternal, fetal, and mechanistic evidence for prenatal CFTR modulator therapy

##### Maternal outcomes in individuals with CF

Across retrospective cohorts, continuation of ETI during pregnancy for individuals with CF is associated with stable or improved lung function, fewer pulmonary exacerbations, improved or stable nutritional status, and deterioration when therapy is discontinued [5].

##### Fetal and neonatal outcomes

###### Gastrointestinal and pancreatic findings

Multiple reports describe resolution or improvement of fetal meconium ileus after maternal initiation of ETI, though not every case of fetal ETI exposure for meconium ileus resolution has been successful [17], (see Table 1).

**TABLE 1:** Published studies reporting outcomes after prenatal CFTR modulator (ETI) exposure.

Author/year	Study type	Population	No. pregnancies	Exposure timing	Key maternal findings	Key fetal/neonatal findings	Safety notes
Nash et al. 2020 [18]	Case series	Pregnant individuals on CFTR modulators	8	Preconception or early pregnancy	Maternal stability preserved	Normal neonatal outcomes	No obstetric or fetal concerns
Fortner et al. 2021 [19]	Case report	Infant prenatally exposed to ETI	1	Throughout pregnancy	Not applicable	False‐negative newborn screen due to low IRT	Reinforces need for confirmatory test
Szentpetery et al. 2022 [9]	Case series	Carrier mothers; fetuses with MI	3	32 weeks	Maternal tolerance of ETI	MI improved;	No adverse fetal events
Collins et al. 2022 [20]	PK/exposure study	Infants with prenatal ETI exposure	2	Throughout pregnancy	Not applicable	Infant ETI levels measurable; normal growth	Rare small cataracts reported
Jain et al. 2022 [21]	Case series	Prenatal ETI exposure	3	In utero	Not discussed	Small, nonvisually significant cataracts	Cataracts rare; mechanism unclear
Ripani et al. 2023 [22]	Maternal–infant PK study	Mothers and infants on ETI	4	Throughout pregnancy	Good maternal tolerance	Confirmed fetal ETI exposure; normal neonatal outcomes	No significant complications
Gómez‐Montes et al. 2023 [23]	Case report	Carrier mother; fetus with MI	1	31 weeks	Skin rash with spontaneous resolution	MI resolution, no adverse fetal events	No significant complications
Blumenfeld et al. 2023 [24]	Case report	Carrier mother; fetus with MI	1	Initiated mid‐pregnancy	Good tolerance	MI improved; healthy neonate	No adverse effects
Kowalik et al. 2024 [25]	Case report	Mother with CF continuing ETI, two fetuses with CF	2	Throughout pregnancy	Not applicable	Male infant with preserved vas deferens at birth and 3 months	Infant with gallstones at 3 months; unclear if related to ETI withdrawal
Goralski et al. 2025 [4]	Prospective cohort	Pregnant individuals with CF on ETI	45	Preconception or early	Improved/stable FEV_1_; fewer exacerbations	No anomalies; healthy neonatal outcomes	No major safety concerns
Jain et al. 2025 [5]	Retrospective cohort	Pregnant individuals continuing ETI	32	First or second trimester	Stable lung function; nutritional stability	Healthy neonates	Minimal transient LFT elevations
Chouchana et al. 2025 [6]	National registry/cohort	Mixed CF pregnancies	57	Varied	Maternal health maintained	No increase in congenital anomalies	No fetal safety signal
Metcalf et al. 2025 [8]	Case report	Carrier mother; fetuses with CF and MI	3	After MI diagnosis	Good maternal tolerance	MI improved/resolved in 2; surgery required when ETI started late	No cataracts or anomalies
Bonnel et al. 2025 [10]	Multicenter cohort	Fetuses with CF + MI	7 for treatment of MI; 4 for prophylactic treatment	After MI diagnosis	Maternal tolerance (one person reported rash pruritic)	MI improved in many; surgery required when ETI started late	Timing‐sensitive outcomes; recommended fetal MRI for bowel dilation prior to initiation of ETI
Destoop et al. 2025 [17]	Case report	Carrier mother; fetus with CF and MI	1	27 weeks	Maternal tolerance	MI continued to worsen, required ileostomy; failed case theorized to be related to maternal weight	No adverse effects
Appelt et al. 2025 [26]	Case report	Mother with CF, fetus with CF	1	From conception	Not applicable	Maintenance of pancreatic function through 8 months with in utero and breast milk exposure, then switched to direct ETI	No adverse events
Haskett et al. 2025 [27]	Case series	Mixed carrier mothers and mothers with CF; all infants with CF, some with MI	4	Variable	Not discussed	Elevated fetal lipase after withdrawal of ETI exposure	Possible pancreatic injury after withdrawal of ETI exposure, may be an indication for direct treatment of infant with CF

Abbreviations: CF, cystic fibrosis; CFTR, CF transmembrane conductance regulator; ETI, elexacaftor/tezacaftor/ivacaftor; FEV_1_, forced expiratory volume in 1 s; IRT, immunoreactive trypsinogen; LFT, liver function test; MI, meconium ileus.

Some infants exposed to ETI from conception demonstrate preserved exocrine pancreatic function despite CF genotypes traditionally associated with pancreatic insufficiency. However, long‐term evaluation is essential to confirm sustained pancreatic preservation, as fecal elastase levels may decline to insufficiency levels (<200 µg/g) after birth if maternal CFTR modulator exposure stops and the infant is not directly dosed with CFTR modulator [27].

Continued ETI exposure through breastfeeding may help maintain pancreatic function. Four infants with prenatal ETI exposure developed elevated serum lipase—a marker of pancreatic cell injury—as ETI exposure diminished postnatally, suggesting that ongoing postnatal ETI therapy is likely necessary to prevent progressive pancreatic damage [27]. The relationship between CFTR function restoration (measured by sweat chloride), pancreatic function (measured by fecal elastase), and pancreatic injury (measured by serum lipase) underscores the importance of serial monitoring in ETI‐exposed infants and highlights a potential role for early postnatal ETI therapy in prenatally exposed infants.

False‐negative newborn screening results due to lower circulating immunoreactive trypsinogen (IRT) levels have been documented in infants with prenatal ETI exposure [15, 28].

###### Male reproductive tract

The only documented case of vas deferens preservation occurred in two male infants with CF whose mother, also affected by CF, took ETI throughout pregnancy [25]. This finding is particularly significant given that approximately 98%–99% of males with CF develop congenital bilateral absence of the vas deferens (CBAVD) due to in utero destruction of the reproductive tract. Studies of CF fetuses demonstrate that vas deferens development appears normal until approximately 22 weeks of gestation, after which focal inflammatory changes and obstructive processes lead to progressive duct atresia [29]. ETI exposure through a carrier mother may occur too late in gestation to prevent this developmental disruption, as the critical window for vas deferens preservation likely occurs in the first or early second trimester when CFTR‐mediated secretory dysfunction begins to cause luminal obstruction [30].

###### Cataracts

Non‐CF neonatal rats that were directly dosed with ivacaftor developed cataracts, leading to a warning in the prescribing label for pediatric patients for all ivacaftor‐containing products. CF rat and ferret models have not developed cataracts following in utero CFTR modulator exposure [31, 32]. Small, nonvisual significant cataracts have been reported following prenatal ETI exposure [21, 33], though subsequent cohorts have not consistently reproduced this finding. Newborn ophthalmologic evaluation is recommended.

The hypothesized benefits of prenatal CFTR modulator are summarized in Table 2.

**TABLE 2:** Hypothesized benefits of prenatal CFTR modulator therapy by organ system and gestational timing.

Organ system	Pathology begins	Potential benefit of prenatal ETI/VTD
Pancreas	Early‐mid‐2nd trimester	• Increase ductal fluid secretion • Reduce acinar destruction • Potential preservation of exocrine function
Intestine (meconium ileus)	Mid‐2nd trimester onward	• Increase luminal hydration • Reduce meconium viscosity • Improve peristalsis • Prevent or improve MI
Airways/lungs	Early 2nd trimester	• Increase airway surface liquid • Reduce mucus plugging • Limit airway remodeling
Hepatobiliary system	Mid‐2nd trimester	• Improve bile fluidity • Reduce inspissation and cholestasis
Male reproductive tract	Early fetal life	• Possible reduction in severity of vas deferens degeneration (prevention unlikely once established)
Growth/nutrition	Throughout pregnancy	• Improve placental nutrition via maternal health stabilization

Abbreviations: CFTR, cystic fibrosis transmembrane conductance regulator; ETI, elexacaftor/tezacaftor/ivacaftor; VTD, vanzacaftor/tezacaftor/deutivacaftor.

##### Mechanistic basis for fetal benefit

Potential, yet unproven, fetal impacts of CFTR modulator include increased hydration of pancreatic and biliary secretions, decreased meconium viscosity, enhanced intestinal motility, improved airway fluid balance, and mitigation of early hepatobiliary obstruction as illustrated in Figure 1.

**FIGURE 1:**
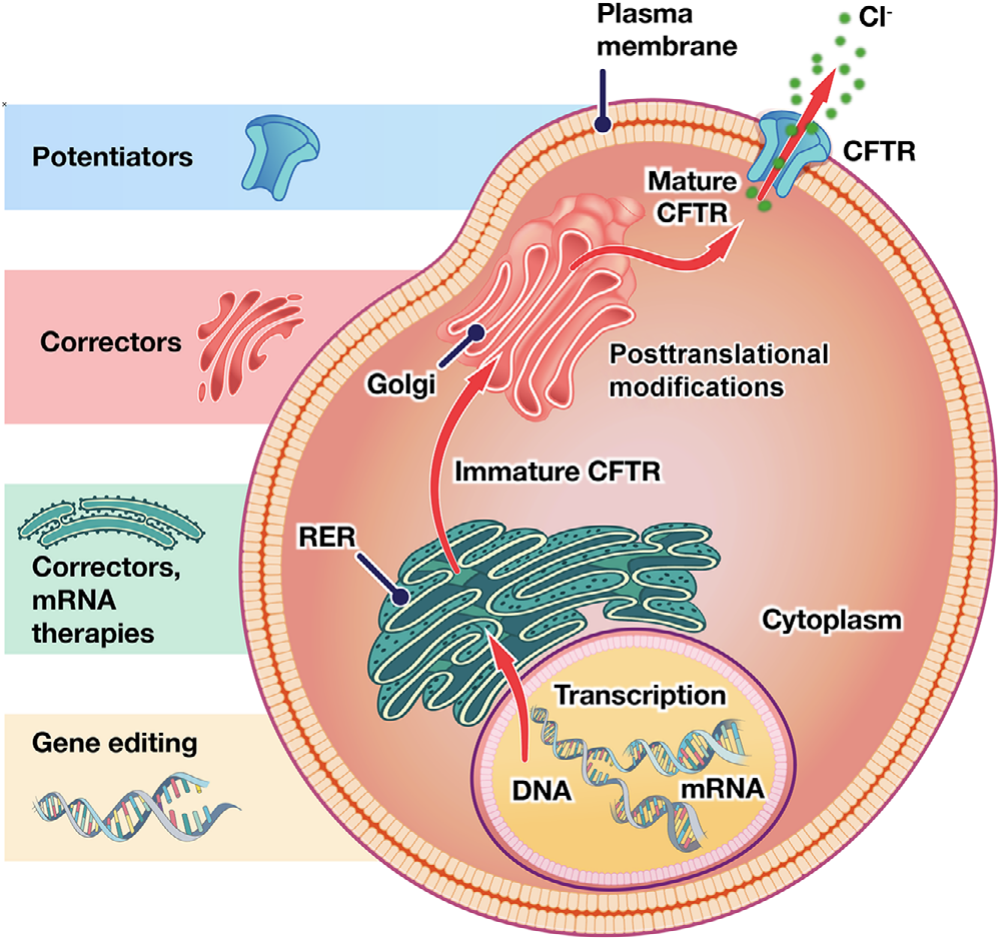
Mechansim of action. CFTR, cystic fibrosis transmembrane conductance regulator. RER, Rough Endoplasmic Reticulum.

##### Placental transfer and fetal exposure

Although limited, some pharmacokinetic studies have demonstrated that ETI components are present in cord blood at concentrations similar to or exceeding maternal levels [22]. Currently, there are no published human data on the effects of vanzacaftor or deutivacaftor in pregnancy. Because these molecules are structurally slightly different, they cannot be assumed to have identical side effects or safety profiles in pregnancy. Animal reproductive models show no dose‐limiting toxicity at human‐equivalent doses, although CF rodent data raise concerns regarding fetal brain accumulation, the human relevance of which remains unclear [34] (Figure 2).

**FIGURE 2:**
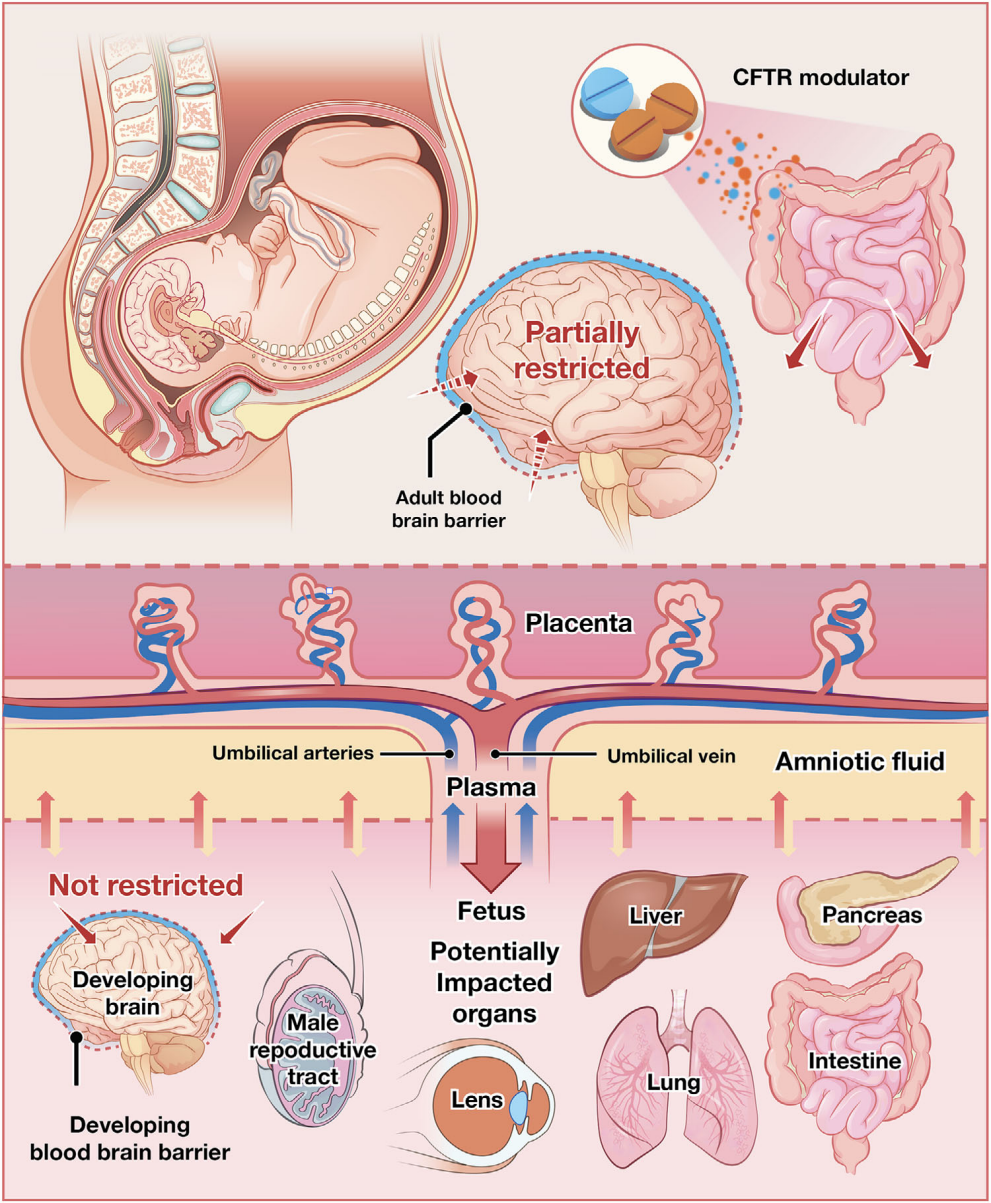
Placental transfer. CFTR, cystic fibrosis transmembrane conductance regulator.

## 4 SAFETY OF PRENATAL CFTR MODULATOR EXPOSURE

ETI and VTD are generally well tolerated. Rash, rhinorrhea, headache, abdominal pain, diarrhea, and transient liver enzyme elevation occur in a minority of cases [5, 6]. Consideration should be given to counseling pregnant patients initiating CFTR modulator—particularly for fetal indications—regarding potential changes in mood or mental health, recognizing that such symptoms may be multifactorial during pregnancy and treatment initiation.

Data from over 150 pregnancies exposed to ETI show no increased risk of major congenital anomalies, preterm delivery, or miscarriage compared to expected rates, with reassuring neonatal outcomes, with one large cohort demonstrating a significantly lower rate of small‐for‐gestational‐age infants (6.8% vs. 16.1% in unexposed pregnancies with CF), and 78% of deliveries occurring at term. There have been no stillbirths or neonatal deaths reported in the largest case series to date [6, 35, 36, 37].

While multiple reports describe resolution or improvement of fetal meconium ileus after maternal ETI initiation, one case experienced increased bowel dilation after ETI introduction, with MRI revealing intestinal atresia [9]. The MODULE CF study protocol recommends a one‐time fetal MRI to assess for potential complications such as worsened bowel dilation or volvulus, particularly in severe cases.

Although rare, hepatotoxicity is among the most common adverse effects of CFTR modulator and requires monitoring in both mothers and exposed infants. Elevated liver enzymes occur in approximately 6%–8% of patients on ETI, with ivacaftor contributing most significantly through reactive oxygen species production and mitochondrial dysfunction [38]. While severe hepatotoxicity (ALT and/or AST > 5× the upper limit of normal with bilirubin > 2× the upper limit of normal) is rare (0.9% in one large cohort), liver function tests should be monitored in pregnant individuals taking ETI and in newborn infants with prenatal exposure [39].

Individuals with mild chronic liver disease (e.g., nonalcoholic fatty liver disease) may still be candidates for therapy with careful monitoring, whereas patients with advanced liver dysfunction, cirrhosis, or significant baseline transaminase elevation should undergo individualized risk–benefit assessment in consultation with hepatology. Limited data exist regarding use in patients with hepatic adenomas; therefore, multidisciplinary evaluation is recommended.

If liver enzyme elevations occur during therapy, repeat laboratory testing and clinical assessment are recommended to determine severity and trajectory. Mild asymptomatic elevations (e.g., <3× the upper limit of normal may be monitored with closer follow‐up. For moderate elevations (3–5× the upper limit of normal), clinicians may consider increased laboratory surveillance, dose interruption, or multidisciplinary reassessment depending on maternal clinical status and fetal indication.

Discontinuation of therapy should be considered for significant hepatotoxicity, typically defined as transaminases ≥5× or ≥3× the upper limit of normal with accompanying bilirubin elevation or clinical symptoms. In such cases, prompt evaluation and coordination with CF specialists and hepatology are recommended to determine whether therapy can be safely resumed or should be permanently discontinued.

The FDA approved VTD in December 2024, and there is no substantial real‐world data to comment on fetal safety. As with ETI, animal reproductive models indicate no fetal harm at normal human doses, but there are no published real‐world data in humans to comment on fetal safety.

## 5 PRENATAL THERAPY FOR FETUSES WITH CF

Referral to a multidisciplinary center with expertise in CF and MFM should be considered when prenatal CFTR modulator therapy is being discussed, as access to transplacental therapy and coordinated maternal‐fetal management may be limited to specialized programs. Ideally, care should involve collaboration between MFM specialists, CF pulmonologists and gastroenterologists, genetic counselors, neonatologists, and pediatric subspecialists. However, some centers may not have specific expertise in CF. In such situations, consultation with regional CF centers or collaboration with institutions experienced in CFTR modulator therapy should be considered.

### 5.1 Rationale

Because a substantial amount of CF pathology occurs during fetal development, prenatal CFTR modulation may modify disease onset and progression before irreversible damage occurs.

### 5.2 Clinical experience

Case series, and the prospective MODUL‐CF multicenter study, report improvement or resolution of meconium ileus when ETI/VTD is initiated during pregnancy in most cases but not all. Earlier initiation appears more effective; late initiation (≥30–36 weeks) may be less likely to prevent the need for medical or surgical intervention following birth. That said, the data are limited, and it is unclear whether the differences seen are related to the time needed to see sonographic improvement, generally on the order of weeks, versus biological limitations of the medications at later gestational ages or when the meconium ileus is more severe [37].

### 5.3 Dosing and prescription

Dosing for prenatal CFTR modulator follows standard adult regimens. For ETI, the dose is elexacaftor 200 mg/tezacaftor 100 mg/ivacaftor 150 mg in the morning and ivacaftor 150 mg in the evening, administered orally. For VTD, standard dosing is vanzacaftor 20 mg/tezacaftor 100 mg/deutivacaftor 250 mg administered orally once daily. These medications should be taken with fat‐containing food to optimize absorption.

At the institutions of this review's authors, prescriptions for prenatal CFTR modulator are managed through CF centers in collaboration with MFM specialists. Published cases of prenatal therapy have been reported by multidisciplinary teams including CF specialists, MFM physicians, neonatologists, pulmonologists, gastrointestinal (GI) specialists, and genetic counselors.

### 5.4 Genetic counseling framework

Referral to a multidisciplinary center with expertise in CF and MFM should be considered when prenatal CFTR modulator therapy is being discussed, as access to transplacental therapy and coordinated maternal‐fetal management may be limited to specialized programs.

*Counseling to discuss prenatal modulator therapy is indicated when*:

- Fetal CF with two disease‐causing variants, one of which is responsive to modulators, is confirmed
- Ultrasound identifies bowel echogenicity and dilation, suggestive of evolving meconium ileus
- Both parents are CF carriers
- The pregnant individual has CF and is using a CFTR modulator, and/or
- Expectant parents would like to learn more about prenatal therapy for an affected CF fetus.

The American College of Obstetricians and Gynecologists (ACOG) recommends offering CF carrier screening to all pregnant individuals [40].

Advances in sequencing‐based diagnostic approaches, including next‐generation sequencing panels and full CFTR gene sequencing, have substantially improved the detection of pathogenic variants compared with targeted genotyping panels. This is particularly important in individuals from non‐European populations, in whom standard variant panels may miss disease‐causing mutations. Comprehensive sequencing strategies therefore improve diagnostic yield and reduce the likelihood of false reassurance when fetal disease is suspected.

Cell‐free DNA–based single‐gene screening for CF is increasingly available and may identify pregnancies at risk for fetal CF earlier in gestation. Positive screening results require confirmatory diagnostic testing with chorionic villus sampling or amniocentesis, and negative screening does not exclude disease. However, therapy may be considered on a case‐by‐case basis when confirmation is pending or not feasible, following multidisciplinary discussion and counseling regarding uncertainty. Counseling should also address implications for newborn screening. Prenatal exposure to CFTR modulators may reduce IRT levels and lead to false‐negative newborn screening results. Therefore, infants with known or suspected prenatal exposure should undergo confirmatory diagnostic testing regardless of newborn screening outcomes.

### 5.5 Eligibility for prenatal CF therapy for fetal indications

Prenatal CFTR modulator for fetal benefit requires a carefully defined candidate profile, given the investigational nature of treatment and the variability in fetal response. As summarized in Table 3, eligibility is restricted to pregnancies with confirmed fetal CF by diagnostic genetic testing (chorionic villous sampling or amniocentesis) with or without fetal bowel dilation suggestive of meconium ileus, ideally during a gestational window in which therapy may alter disease progression, and in settings where maternal safety, fetal surveillance capacity, and multidisciplinary expertise are assured. Conversely, prenatal therapy should not be routinely offered when fetal CF is unconfirmed, neither CFTR variant is responsive to available modulators, alternative etiologies explain bowel findings, or therapy would begin too late to meaningfully influence outcomes. In cases with ultrasound findings consistent with meconium ileus and a positive fetal single‐gene noninvasive prenatal screen (sg‐NIPS) result suggestive of CF, diagnostic confirmation is strongly encouraged; however, therapy may be considered on a case‐by‐case basis when confirmation is pending or not feasible, following multidisciplinary discussion and counseling regarding uncertainty. Moreover, the utility of therapy for the prevention of meconium ileus, or preservation of pancreatic function, or rescue of reproductive function in males in cases of fetal CF but with normal appearing bowel, particularly in the second trimester, remains unknown. These criteria support consistent, individualized decision‐making as clinical experience evolves.

**TABLE 3:** Suggested eligibility criteria for prenatal CFTR modulator therapy.

Category	Eligibility criteria
Criteria for offering prenatal CF therapy	Confirmed fetal CF by diagnostic genetic testing (CVS or amniocentesis), *with or without* bowel dilation. *Important*: Recent reports show false‐negative CF results with NIPS [41]. * In cases with ultrasound findings suggestive of evolving MI (dilated, echogenic bowel) and a positive fetal single‐gene NIPS result suggestive of CF, diagnostic confirmation is strongly encouraged; however, therapy may be considered on a case‐by‐case basis when confirmation is pending or not feasible, following multidisciplinary discussion and counseling regarding uncertainty.
	At least one modulator‐responsive CFTR variant (ETI/VTD responsive).
	Gestational age early enough for potential benefit, ideally before ∼30–32 weeks.
	Maternal suitability for ETI/VTD, including no contraindications and ability to complete LFT monitoring.
	Capacity for structured fetal surveillance, including serial ultrasounds.
	Multidisciplinary team involvement, including MFM, CF specialists, neonatology, pediatric GI, and genetics.
	Documented shared decision‐making, acknowledging investigational use, uncertain long‐term outcomes, and variable response based on timing.
Non‐eligibility situations	Fetal CF not confirmed by diagnostic testing.
	CFTR variants are non‐modulator responsive.
	Ultrasound suggests alternative diagnoses (e.g., volvulus, atresia without CF, other non‐CF etiologies).
	Initiation would occur too late to alter fetal course (typically ≥36 weeks).
	Maternal contraindications to therapy or inability to comply with monitoring.
	Family declines investigational therapy or cannot participate in required follow‐up.

Abbreviations: CF, cystic fibrosis; CFTR, CF transmembrane conductance regulator; CVS, chorionic villus sampling; ETI, elexacaftor/tezacaftor/ivacaftor; GI, gastroenterology; IRT, immunoreactive trypsinogen; LFT, liver function test; MFM, maternal‐fetal medicine; MI, meconium ileus; NBS, newborn screening; NIPS, noninvasive prenatal testing; VTD, vanzacaftor/tezacaftor/deutivacaftor.

#### 5.5.1 Maternal‐fetal and neonatal monitoring

A suggested monitoring protocol is outlined in Table 4, but in brief, consider performing liver function tests every 4–8 weeks, ultrasounds every 2–4 weeks, coordinating CFTR modulator therapy in consultation with CF specialists, coordinating delivery at a center with appropriate neonatal and pediatric services that can manage neonates with CF, and arranging a neonatal ophthalmologic evaluation.

**TABLE 4:** Suggested maternal and fetal monitoring schedule during prenatal CFTR modulator therapy.

Time point	Maternal monitoring	Fetal monitoring	Neonatal planning
Baseline (before or at pregnancy confirmation)	• LFTs • FEV_1_ and pulmonary assessment • Nutritional status • Review ETI/VTD regimen	• Detailed anatomy scan • Assess bowel (echogenic bowel, MI, dilation)	• Notify CF team • Plan for ophthalmologic evaluation of infant
Every 4–8 weeks	• LFTs • Symptoms, weight, exacerbations	• Ultrasound q2–4 weeks for bowel findings, growth	—
If MI present	• Same as above	• Ultrasound q1–2 weeks • Consider fetal MRI if severe/borderline	• Prepare pediatric GI and neonatology teams
Third trimester	• Assess maternal stability • Repeat LFTs	• Growth assessment • Bowel reassessment	• Delivery planning with CF, GI, NICU
Antenatal surveillance		Follows routine obstetrics practices	
Birth	Follows routine obstetrics practices	—	• Ophthalmologic exam • Sweat chloride test • Fecal elastase • Interpret NBS with caution—LFTs, CBC, coagulation studies, amylase, lipase per PROTECT workshop
Postpartum	• Evaluate breastfeeding plan	—	• Early CF clinic visit • Repeat fecal elastase measures • Monthly LFTs and GGT with ongoing breastmilk per PROTECT workshop

Abbreviations: CBC, complete blood count; CF, cystic fibrosis; ETI, elexacaftor/tezacaftor/ivacaftor; FEV_1_, forced expiratory volume in 1 s; GI, gastrointestinal; GGT, gamma‐glutamyl transferase; LFT, liver function test; MI, meconium ileus; MRI, magnetic resonance Imaging; NICU, neonatal intensive care unit; PROTECT, PRenatal MOdulator Treatment to PrEvent CF ComplicaTions; VTD, vanzacaftor/tezacaftor/deutivacaftor.

In the absence of evidence demonstrating increased risks of stillbirth, placental insufficiency, or fetal compromise related to CFTR modulator exposure, most experts recommend that antenatal testing and delivery timing follow standard obstetric indications. Decisions regarding initiation of nonstress testing, biophysical profile surveillance, or early delivery should therefore be guided by routine maternal or fetal conditions rather than prenatal modulator exposure alone. As additional clinical experience accumulates, future studies may better define whether specific surveillance strategies or delivery considerations are warranted.

#### 5.5.2 Breastfeeding considerations

*ETI and its metabolites transfer through breast milk at concentrations lower than maternal plasma, with measurable but low levels detected in breastfed infant plasma*. While these levels appear subtherapeutic, emerging evidence suggests potential clinical benefits in some breastfed infants with CF, including lower sweat chloride values and sustained pancreatic sufficiency in those with prenatal exposure. One case documented pancreatic sufficiency attainment in an infant whose carrier mother initiated ETI postpartum specifically to provide indirect exposure via breastfeeding [20, 22, 27, 42].

*In individuals with CF, maternal continuation of ETI postpartum is recommended to maintain maternal pulmonary function and reduce postpartum exacerbations*. However, international practice varies: some centers support breastfeeding with maternal ETI use, while others recommend against it due to uncertainties regarding long‐term safety.

*Direct CFTR modulator administration to young infants is occurring off‐label at some CF centers*, particularly when breast milk levels are insufficient to achieve therapeutic plasma concentrations. Ivacaftor is FDA‐approved for infants as young as 1 month with gating mutations, lumacaftor‐ivacaftor is effective in infants and young children homozygous for the F508del variant and approved down to 1 year of age, and clinical trials for ETI in children 12–24 months are ongoing [43, 44].

*Prenatal and lactational ETI exposure may lower IRT, increasing the likelihood of false‐negative newborn screening;* therefore, all ETI‐exposed infants at risk for CF should undergo confirmatory testing, including genetic testing, sweat chloride testing, and fecal elastase, regardless of newborn screening results.

*Standard CF newborn care should begin immediately*, including pancreatic enzyme replacement therapy (PERT), nutritional optimization, salt supplementation, and airway clearance.

*Infants with modulator‐responsive genotypes should be considered for direct modulator initiation once they reach the approved age threshold*, particularly if breastfeeding exposure alone is insufficient to maintain clinical benefits.

*A neonatal ophthalmologic exam is recommended for any infant with prenatal or lactational ETI exposure* due to rare reports of small, nonvisually significant cataracts, though only limited formal evaluations have been performed to date.

## 6 ETHICAL, REGULATORY, AND ACCESS CONSIDERATIONS

Prenatal CFTR modulator therapy for fetal benefit remains investigational, and its use raises several ethical considerations. Although early case series suggest potential fetal benefit—particularly in cases of evolving meconium ileus—evidence remains limited, and optimal timing, dosing duration, and long‐term outcomes are not yet established. For this reason, some experts recommend that intentional prenatal therapy for fetal indications be offered preferentially within research protocols or prospective registries whenever possible. However, given the rarity of affected pregnancies and the biological plausibility of benefit, carefully selected off‐protocol clinical use may be considered in specialized centers following multidisciplinary review and detailed counseling.

Discussions should include discussion of the investigational nature of therapy, uncertainty regarding fetal and long‐term neurodevelopmental outcomes, potential maternal adverse effects, including hepatotoxicity, and the possibility that treatment may not prevent complications such as meconium ileus or pancreatic insufficiency. Counseling should also address downstream implications for newborn screening and postnatal management.

Insurance coverage for carrier mothers is not guaranteed and may be delayed, or copays may be cost‐prohibitive.

Delivery and postdelivery planning are important components of care. Because infants exposed to prenatal CFTR modulator may require specialized neonatal evaluation—including confirmatory CF testing, ophthalmologic screening for cataracts, and assessment of pancreatic function—delivery at or near a center with neonatal intensive care capabilities and access to pediatric pulmonology or CF specialists should be considered when feasible. Given the lack of approval of CFTR modulator in infancy for ETI/VTD outlined above, a plan for treating the infant postdelivery should be established to prevent potential withdrawal syndrome.

## 7 FUTURE DIRECTIONS

Current and future prenatal ETI/VTD registries should incorporate serial fetal imaging, cord blood drug level measurements, and detailed neonatal as well as long‐term outcome data. Adaptive study designs, particularly those focused on meconium ileus–specific cohorts, are likely to be the most feasible and informative approaches in this evolving field.

Further research is needed to clarify fetal drug metabolism, placental transport mechanisms, and tissue‐specific effects. The development and integration of biomarkers will be essential, with potential tools including fetal MRI bowel assessments during pregnancy, as well as neonatal fecal elastase, IRT, and sweat chloride concentrations in infants with CF.

Long‐term follow‐up remains critical, with key outcomes including pulmonary trajectory, maintenance of pancreatic function, growth, GI morbidity, and neurodevelopment.

## 8 CONCLUSION

Prenatal CFTR modulator therapy is an emerging frontier in molecular fetal medicine. Maternal continuation to treat maternal CF appears safe and beneficial, and early evidence indicates potential fetal benefit in selected circumstances, particularly for meconium ileus. Knowledge gaps remain, and therapy for fetal indications is investigational. MFM and CF specialists must provide nuanced counseling, structured monitoring, and interdisciplinary care while supporting ongoing research to define therapeutic windows, long‐term safety, and standardized protocols.

## CONFLICT OF INTEREST STATEMENT

A.M. reports funding from the Cystic Fibrosis Foundation and the National Institutes of Health. J.E.H. reports funding from the Cystic Fibrosis Foundation and consulting fees from Vertex Pharmaceuticals. I.S.G. reports funding from Vaincre La Mucoviscidose, Cystic Fibrosis Foundation and educational fees from Vertex Pharmaceuticals. E.T.Z. reports funding from the Cystic Fibrosis Foundation, funding for clinical trials from Vertex Pharmaceuticals, and consulting fees from Boehringer‐Ingelheim. S.E.S. reports funding from the Cystic Fibrosis Foundation and has served as primary investigator for clinical trials from Vertex Pharmaceuticals (all funds paid to her institution). A.T.T. reports funding from the Cystic Fibrosis Foundation and the Boomer Esiason Foundation and has served as primary investigator for clinical trials from Vertex Pharmaceuticals (all funds paid to his institution). Unrelated to this work, as faculty at an institution that is part of the CF Therapeutics Development Network, he is or has been site Primary Investigator (PI) on studies for Recode Therapeutics. J.L.T.C., as the Director of the Cystic Fibrosis (CF) Therapeutics Development Center at National Jewish Health, has been primary investigator on studies for Vertex Pharmaceuticals and has done clinical trial design advising for Vertex Pharmaceuticals (all funds paid to her institution). Unrelated to this work, as faculty at an institution that is part of the CF Therapeutics Development Network, she is or has been site/national PI on studies for 4DMT, Splisense, Recode, and Sionna. She has done clinical trial consulting and/or advisory boards for Kither Biotech and 4DMT and served as a DMC Chair for AbbVie. She receives grant funding from the CF Foundation and NIH. She serves as the adult patient care representative to the CFF Board of Trustees, and on the CF Foundation's Clinical Research Executive Committee, Clinical Research Advisory Board, and as immediate past chair of the CF TDN's Sexual Health, Reproduction and Gender Research‐Working Group, as previous Chair of the Health Science Team Awards study section, and on the scientific advisory board for Emily's Entourage. She is the Chair for the ATS International Conference Committee and is a member of the International Advisory Board for Lancet Respiratory Medicine and the Editorial Board for the Journal of Cystic Fibrosis. S.D.S. reports funding from the Cystic Fibrosis Foundation and the National Institutes of Health. E.B.B. reports funding from the Cystic Fibrosis Foundation and the National Institutes of Health. The other authors declare no conflicts of interest.

## FUNDING INFORMATION

The authors received no specific funding for this work.

## Data Availability

Data sharing is not applicable to this article as no datasets were generated or analyzed during the current study.
